# Effectiveness of oral antivirals in reducing adverse outcomes in asthmatic patients with non-severe COVID-19: A multi-institutional retrospective cohort study

**DOI:** 10.1097/MD.0000000000048378

**Published:** 2026-04-17

**Authors:** Shao-Chi Fang, Ya-Wen Tsai, Chien-Ming Chao, Chih-Cheng Lai

**Affiliations:** a Department of Radiology, Kaohsiung Medical University Chung-Ho Memorial Hospital Center, Kaohsiung, Taiwan; b Center for Integrative Medicine, Chi Mei Medical Center, Tainan City, Taiwan; c Department of Intensive Care Medicine, Chi Mei Medical Center, Tainan City, Taiwan; d Department of Dental Laboratory Technology, Min-Hwei College of Health Care Management, Tainan City, Taiwan; e Department of Intensive Care Medicine, Chi Mei Hospital, Tainan City, Taiwan.

**Keywords:** asthma, COVID-19, molnupiravir, nirmatrelvir/ritonavir, SARS-CoV-2

## Abstract

Preventing the progression of coronavirus disease 2019 (COVID-19) is a critical issue, particularly in patients with comorbidities such as asthma. However, real-world data on the effectiveness of oral antivirals in patients with asthma and non-severe COVID-19 are limited. This study was conducted to evaluate the effectiveness of 2 oral antivirals, nirmatrelvir/ritonavir (NMV-r) and molnupiravir, in reducing adverse outcomes in asthmatic patients with non-severe COVID-19. This retrospective cohort study included non-hospitalized adults with asthma who were diagnosed with COVID-19 between January 1, 2022, and March 16, 2024, using data from the TriNetX Research Network. The study group received NMV-r or molnupiravir; the control group received no antivirals. Covariates were balanced between the groups using propensity score matching. The primary outcome was the composite incidence of all-cause mortality, all-cause hospitalization, and mechanical ventilation use within 30 days. After matching, each group comprised 19,235 patients. The study group showed significantly lower composite primary outcome (hazard ratio [HR]: 0.57; 95% confidence interval [CI]: 0.49–0.66). Specifically, the study group was associated with significantly lower risk of all-cause hospitalization (HR: 0.59; 95% CI: 0.50–0.69) and all-cause mortality (HR: 0.34; 95% CI: 0.16–0.73) and mechanical ventilation requirements (HR: 0.08; 95% CI: 0.01–0.58). Significantly risk reduction was also observed in subgroups including both sexes, different ages, those with hypertension, dyslipidemia, diabetes mellitus, those receiving NMV-r, and those who had received less than 1 dose and more than 3 doses of vaccine. Our findings suggest oral antivirals, particularly NMV-r, reduce adverse outcomes in asthmatic patients with non-severe COVID-19.

## 1. Introduction

Coronavirus disease 2019 (COVID-19), caused by the severe acute respiratory syndrome coronavirus 2 (SARS-CoV-2), has affected approximately 776 million people globally.^[1]^ Since its outbreak in 2019, it has been deemed a major public health issue.^[2,3]^ In most people with COVID-19, the infection does not develop into severe COVID-19.^[4]^ The annualized incidence of COVID-19 and severe COVID-19 was estimated at 13.9% (95% confidence interval [CI]: 13.3–14.4%) and 2.0% (95% CI: 1.8–2.2%), respectively.^[5]^ Due to the high morbidity and mortality associated with severe COVID-19, preventing disease progression, particularly for high-risk patients, is a critical issue.^[4]^

Asthma, a chronic inflammation involving the lower respiratory tract, affects nearly 400 million people worldwide.^[6–8]^ Asthma and many underlying medical conditions, including diabetes mellitus, cardiovascular disease, chronic obstructive pulmonary diseases, or chronic kidney diseases, have been identified as risk factors for COVID-19 progression.^[9–13]^ For ambulatory patients with mild-to-moderate COVID-19 at high risk for progression to severe disease, oral antivirals, including nirmatrelvir/ritonavir (NMV-r) or molnupiravir (MOV), have shown promising clinical benefit and have been recommended.^[4,14,15]^ Based on the risk of developing severe COVID-19, the World Health Organization classified patients into 3 categories, with chronic cardiopulmonary diseases considered a moderate-risk group.^[16]^ However, World Health Organization guidelines do not specify whether asthma belongs to chronic cardiopulmonary diseases, leading to uncertainty regarding oral antiviral use in patients with asthma. In contrast, the US Centers for Disease Control and Prevention have identified moderate-to-severe asthma as a risk factor for developing severe COVID-19 symptoms and recommends oral antivirals for this vulnerable group.^[13]^ Taiwan’s guidelines also suggest antivirals for patients with asthma comorbid with non-severe COVID-19 conditions.^[17]^ However, real-world data on the effectiveness of oral antivirals in patients with asthma and non-severe COVID-19 are limited. Hence, this study aimed to utilize real-world data to demonstrate the importance of oral antivirals in this patient group.

## 2. Methods

### 2.1. Data source

This retrospective cohort study utilized data sourced from the TriNetX Research Network, an extensive collaborative platform for clinical research in global health. The network compiles real-time electronic medical records obtained from a vast pool of 130 million patients across 92 healthcare organizations (HCOs). TriNetX adheres strictly to the regulations outlined in the Health Insurance Portability and Accountability Act and other pertinent US federal laws governing the confidentiality and security of healthcare data. The data extraction process from the electronic health record systems of participating institutions is fully conducted to ensure robust assurance before incorporation into the database, following a standardized and systematic format. To protect patient’s privacy, all data are deidentified, with only aggregated counts and statistical summaries provided for the variables of interest. Additionally, TriNetX implements measures to obscure patient counts below 10 to safeguard anonymity.^[18,19]^ The study protocol received approval from the Institutional Review Board of the Chi Mei Medical Center (Approval No.: 11202-002).

### 2.2. Patient selection

We included patients aged ≥18 years who visited HCOs more than 2 times to ensure that these patients had regular follow-up within the HCO, and had a prior diagnosis of asthma (International Classification of Diseases, Tenth Revision, Clinical Modification [ICD-10-CM] code J45) before being diagnosed with COVID-19 between January 1, 2022, and March 16, 2024. Adults with asthma and COVID-19 comorbidities were identified according to the diagnosis codes for COVID-19 (U07.1, J12.81, and J12.82), a positive SARS-CoV-2 RNA test (Logical Observation Identifiers Names and Codes 94309-2, 94500-6, 95406-5, 94502-2, 94565-9, 95608-6, 94759-8, and 94845-5), or positive COVID-19 antigen immunoassay results (Logical Observation Identifiers Names and Codes 94558-4 and 96119-3). Those who received oral antivirals (NMV-r or MOV) were classified as the study group. Conversely, the control group included adults who had not been treated with oral antivirals or remdesivir. To ensure that only non-severe COVID-19 cases were included, this study specifically excluded COVID-19 patients who require initial hospitalization within 5 days after COVID-19 diagnosis or deceased on the day of diagnosis. Additionally, we excluded those were diagnosed with chronic obstructive pulmonary disease [J40-J44]. Covariate data including demographic characteristics (age, sex, and race), socioeconomic status, comorbidities (body mass index [TNX Curated 9083], neoplasms [C00-D49], diabetes mellitus [E08-E13], essential hypertension [I10], hyperlipidemia [E78.5], chronic kidney disease [N18], and ischemic heart disease [I20-I25]), and lifestyle factors (smoking [F17] and alcohol use [F10]), and medication for asthma control (inhaled corticosteroid [ICS]) [anatomical therapeutic chemical, ATC: R03BA], inhaled long-acting beta-2-adrenoreceptoragonists (LABA) [ATC: R03AC], inhaled long-acting muscarinic antagonists (LAMA) [ATC: R03BB], were collected.

### 2.3. Outcomes

The primary outcome was defined as the composite incidence of all-cause mortality, all-cause hospitalization, and mechanical ventilation (MV) use within 30 days following the index date. The secondary outcome encompassed the occurrence of each component individually within the primary outcome. The index date was defined as the date of antiviral prescription in the study group and the diagnosis of COVID-19 in the control group.

### 2.4. Statistical analysis

All statistical analyses were conducted utilizing the TriNetX platform. Baseline characteristics were presented as mean ± standard deviation or frequency (proportion).

Cox proportional hazard regression was employed to compute hazard ratios (HRs) with corresponding 95% CIs. Propensity score matching (PSM) was conducted in a 1:1 ratio using the TriNetX built-in platform, utilizing the greedy nearest-neighbor algorithm for covariate adjustment. The platform encompasses various software components across different functional areas to ensure accuracy. PSM is a commonly utilized method in observational studies for effectively balancing confounding covariates across treatment groups, thereby facilitating a more precise estimation of treatment effects.^[20]^ A standardized difference of <0.1 was deemed acceptable for achieving a balanced match.

Kaplan–Meier curves were utilized to assess the cumulative probability.^[21]^ A *P* < .05 was considered statistically significant. Pre-specified subgroup analyses were performed based on age, sex, race, comorbidities, asthma severity, type of antivirals, those requiring triple therapy with ICS/LABA/LAMA, and vaccination status. The severity of asthma was categorized into 3 different classes according to ICD-10 codes, including mild (J45.2, J45.3), moderate (J45.4), and severe (J45.5) asthma. Vaccination status was classified into 3 groups, including patients who received 0 to 1 (inadequate vaccination), 2 (full vaccination), and ≥3 vaccine doses (boostered vaccination).

## 3. Results

### 3.1. Patient selection

**Figure 1.**
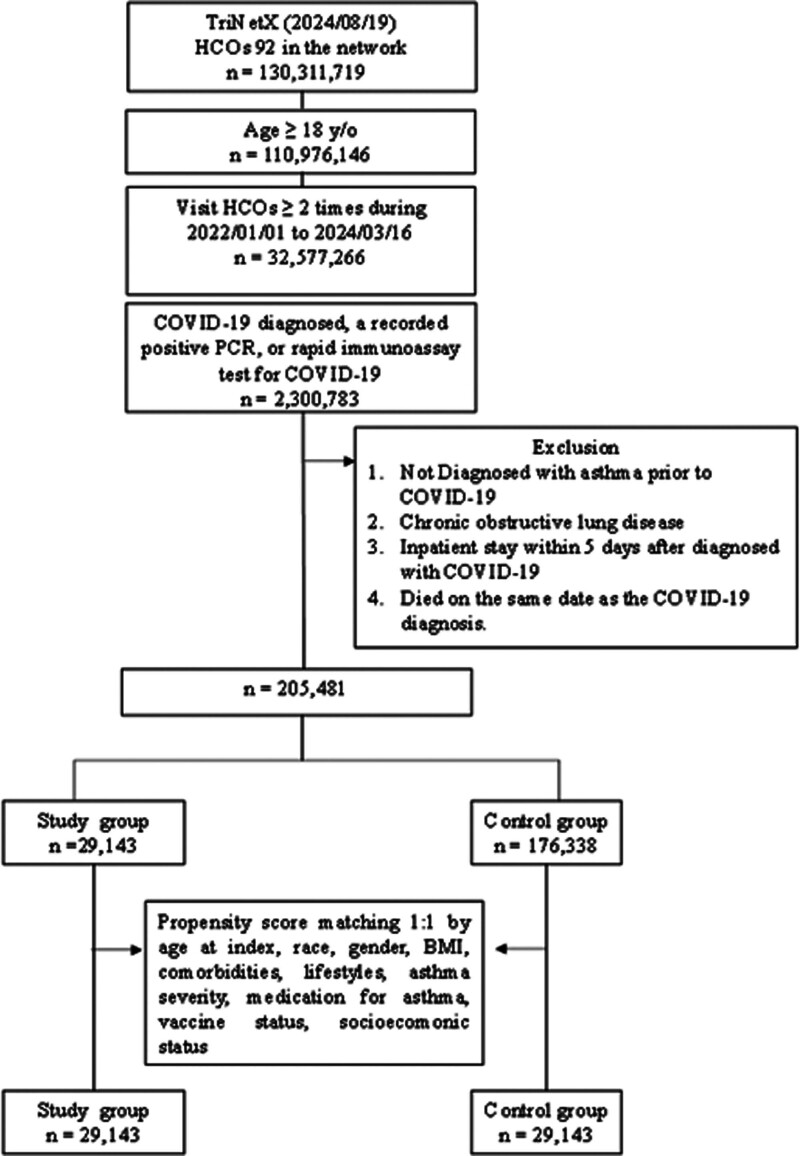
The algorithm of patient selection.

Initially, a total of 130,311,719 patients with available data from 92 HCOs were identified on Augst 19, 2024. Among these, 110,976,146 patients aged ≥ 18 years who made more than 2 visits to HCOs between January 1, 2022, and March 16, 2024, were enrolled. After applying the inclusion and exclusion criteria, 29,143 and 176,338 patients were categorized into the study and control groups, respectively (Fig. 1). Utilizing PSM, the study and control groups each comprised 29,143 individuals.

### 3.2. Demographic characteristics of the included patients

Table 1 presents the baseline characteristics of the study participants in the 2 groups before and after PSM. Before matching, patients in the study group were older than those in the control group. In addition, the study group had a higher prevalence of hypertension, hyperlipidemia, and neoplasms than the control group. The study group had a higher proportion of mild and moderate asthma cases but used more LABA and ICS than the control group. Additionally, a larger percentage of the study group received booster vaccines. After PSM, all the above covariates were well-balanced, with standardized differences <0.1.

**Table 1:** The clinical characteristics of included patients before and after matching.

	Before matching			After matching		
	Oral antiviral group (n = 29,143)	Control group (n = 176,338)	Std diff	Oral antiviral group (n = 29,143)	Control group (n = 29,143)	Std diff
Age at index, years (mean ± SD)	53 ± 16.6	45.9 ± 18.1	0.385	53 ± 16.6	53 ± 17.8	0.004
Sex (%)						
Male	7752 (26.6)	44,965 (27.1)	0.012	7752 (26.6)	7675 (26.3)	0.006
Female	19,710 (67.6)	114,152 (68.9)	0.026	19,710 (67.6)	19,887 (68.2)	0.013
Race (%)						
White	20,848 (71.5)	101,543 (61.2)	0.219	20,848 (71.5)	21,116 (71.5)	0.021
Black or African American	2883 (9.9)	26,978 (16.3)	0.190	2883 (9.9)	2787 (9.6)	0.011
Asian	948 (3.3)	6560 (4.0)	0.038	948 (3.3)	1000 (3.4)	0.010
Social determinants of health associated with adverse outcomes						
Housing and economic circumstances	227 (0.8)	1984 (1.3)	0.045	227 (0.8)	218 (0.8)	0.004
Employment and unemployment	91 (0.3)	825 (0.5)	0.031	91 (0.3)	88 (0.3)	0.002
Education and literacy	22 (0.1)	287 (0.2)	0.029	22 (0.1)	28 (0.1)	0.007
Occupational exposure to risk factos	41 (0.2)	319 (0.2)	0.014	41 (0.2)	36 (0.1)	0.005
Underlying diseases (%)						
Essential hypertension	12,345 (42.4)	56,454 (34.1)	0.172	12,345 (42.4)	12,096 (41.5)	0.017
Hyperlipidemia	8496 (29.2)	35,637 (21.5)	0.177	8496 (29.2)	8387 (28.8)	0.008
Neoplasms	9433 (32.4)	38,680 (23.3)	0.203	9433 (32.4)	9357 (32.1)	0.006
Diabetes mellitus	4955 (17.0)	24,510 (14.8)	0.061	2402 (16.8)	4965 (17.0)	<0.001
Obesity	9467 (32.5)	47,751 (28.8)	0.080	9467 (32.5)	9321 (32.0)	0.011
Ischemic heart diseases	2568 (8.8)	12,507 (7.5)	0.046	2568 (8.8)	2458 (8.4)	0.024
Chronic kidney disease	1543 (5.3)	9248 (5.6)	0.013	1543 (5.3)	1558 (5.4)	0.028
Lifestyle (%)						
Nicotine dependence	1831 (6.3)	14,521 (8.8)	0.094	1831 (6.3)	1756 (6.0)	0.011
Alcohol related disorder	539 (1.9)	3822 (2.3)	0.032	297 (2.1)	468 (1.6)	0.011
Body mass index (mean ± SD)	31.5 ± 8.06	31.3 ± 8.27	0.018	31.5 ± 8.06	31.4 ± 7.97	0.012
Asthma severity						
Mild	13,059 (44.8)	56,465 (34.1)	0.173	13,059 (44.8)	12,839 (44.1)	0.006
Moderate	3952 (13.6)	16,978 (10.2)	0.103	3952 (13.6)	3760 (13.0)	0.019
Severe	503 (1.7)	2704 (1.6)	0.007	503 (1.7)	468 (1.6)	0.009
Medication for asthma						
Inhaled long-acting beta-2 agonist	19,090 (65.5)	91,543 (51.9)	0.200	19,090 (65.5)	19,033 (65.3)	0.004
Inhaled corticosteroid	16,458 (56.5)	75,279 (45.4)	0.223	16,458 (56.5)	16,177 (55.5)	0.019
Inhaled long-acting muscarinic antagonistss	3907 (13.4)	19,379 (11.7)	0.052	3907 (13.4)	3838 (13.2)	0.007
Systemic corticosteroid	362 (1.2)	3201 (1.9)	0.055	362 (1.2)	349 (1.2)	0.004
Vaccine status						
0–1 dose	24,387 (83.7)	150,343 (85.3)	0.026	24,387 (83.7)	24,400 (83.7)	0.002
2 doses	725 (2.5)	14,180 (8.0)	0.022	725 (2.5)	1112 (3.8)	0.007
≥3 doses	4031 (13.8)	11,815 (7.0)	0.138	4031 (13.8)	3631 (12.5)	0.022

### 3.3. Primary and secondary outcomes

**Figure 2.**
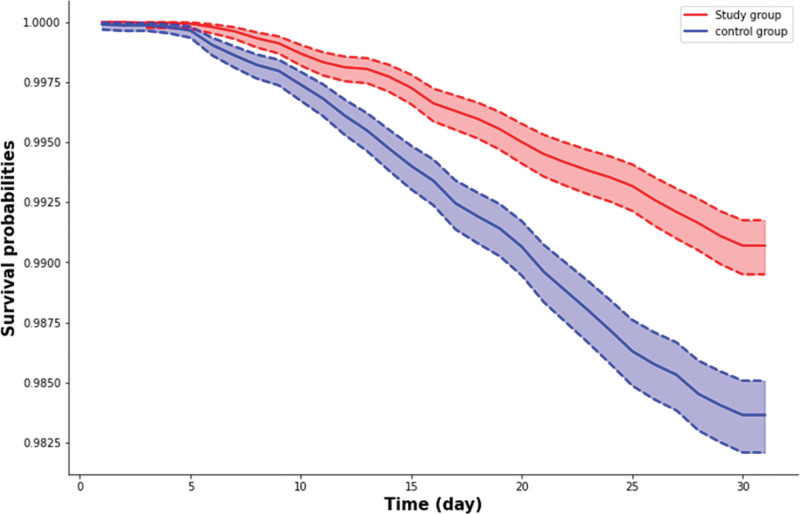
Kaplan–Meier time-to-event free curve for the study group and the control group.

During the 30-day follow-up period, the study group exhibited a reduced composite risk of all-cause mortality, all-cause hospitalization, and MV use compared to the control group (HR: 0.57; 95% CI: 0.49–0.66) (Table 2). The study group showed significantly lower composite primary outcome. Specifically, the study group was associated with significantly lower risk of all-cause hospitalization (HR: 0.59; 95% CI: 0.50–0.69) and all-cause mortality (HR: 0.34; 95% CI: 0.16–0.73) and MV requirements (HR: 0.08; 95% CI: 0.01–0.58) (Table 2). Further Kaplan–Meier curves demonstrated that compared to the control group, the study group had a lower risk of all-cause mortality, all-cause hospitalization, and MV use during the follow-up period (Fig. 2).

**Table 2:** Primary outcome and secondary outcome of study group and control group.

	Patient outcome		Hazard ratio (95% CI)	*P*-value
	Oral antiviral group (n = 29,143)	Control group (n = 29,143)		
Primary outcome	260	453	0.57 (0.49–0.66)	<.0001
Secondary outcome				
Mortality	10	26	0.34 (0.16–0.73)	.003
Admission event	251	420	0.59 (0.50–0.69)	<.0001
Mechanical ventilation	10	13	0.08 (0.01–0.58)	.001

### 3.4. Subgroup analysis

**Figure 3.**
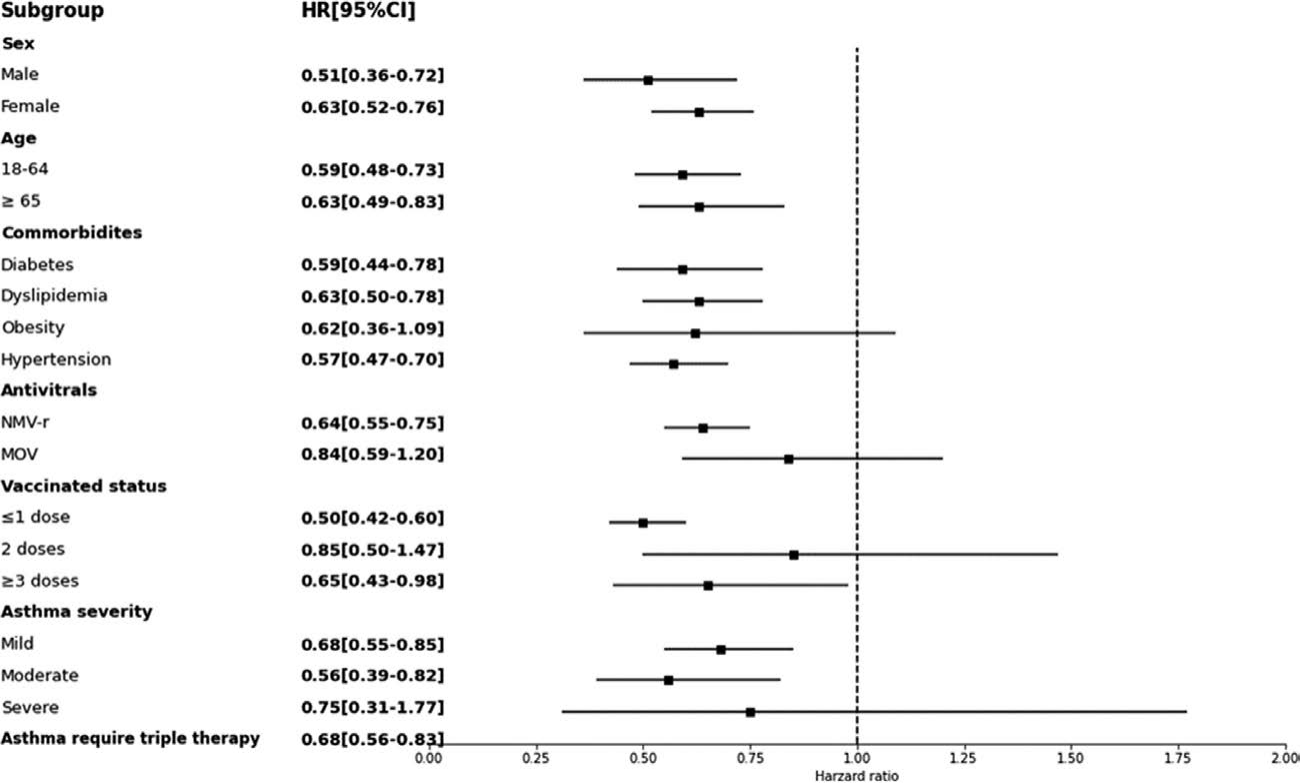
Forest plots of the primary outcome between the study and control group according to the subgroup.

Subgroup analyses were conducted based on age, sex, comorbidities, asthma severity, different antiviral agents, and SARS‐CoV‐2 vaccination status (Fig. 3). Compared to the control group, the study group consistently showed a significantly reduced risk of the primary outcome across 13 0f 17 pre-specified subgroups.

Sex: risk reduction was observed consistently in both male (HR: 0.51; 95% CI: 0.36–0.72) and female (HR: 0.63; 95% CI: 0.52–0.76) patients.

Age: significant differences were observed for patients aged 18 to 64 years (HR: 0.59; 95% CI: 0.48–0.73) and for those aged ≥ 65 years (HR: 0.63; 95% CI: 0.49–0.83).

Comorbidities: significantly lower risks were observed among patients with hypertension (HR: 0.57; 95% CI: 0.47–0.70), diabetes mellitus (HR: 0.59; 95% CI: 0.44–0.78), and dyslipidemia (HR: 0.63; 95% CI: 0.50–0.78).

Vaccination status: risk reduction were significant among people with less than 1 vaccine doses (HR: 0.50; 95% CI: 0.42–0.60) and people with more than 3 vaccine doses (HR: 0.65; 95% CI: 0.43–0.98). However, the risk reduction did not reach statistical significance for those received 2 (HR: 0.85; 95% CI: 0.50–1.47) vaccine doses.

Oral antivirals: compared to the control group, NMV-r treatment was associated with a significant risk reduction (HR: 0.64; 95% CI: 0.55–0.75), whereas MOV treatment was not associated with significant risk reduction (HR: 0.84; 95% CI: 0.59–1.20).

Asthma severity: patients with mild (HR: 0.68; 95% CI: 0.55–0.85) and moderate (HR: 0.56; 95% CI: 0.39–0.82) asthma exhibited significantly lower risks. However, there was no significant risk reduction for patients with severe asthma (HR: 0.75; 95% CI: 0.31–1.77). A significant risk reduction was observed for patients with asthma requiring triple therapy (HR: 0.68; 95% CI: 0.56–0.83).

## 4. Discussion

This large retrospective cohort study found that patients with asthma who received oral antiviral therapy were associated with a lower risk of COVID-19 adverse outcomes than controls. Similar trends remained consistent across most of subgroup analyses for age, sex, comorbidities, severity of asthma, different antiviral use, and SARS‐CoV‐2 vaccination status, despite some differences did not reach statistical significance. Our study findings were consistent with those of previous reports. A retrospective study conducted in Hong Kong investigated the effectiveness of MOV and NMV-r in unvaccinated patients with chronic respiratory diseases, including asthma, chronic obstructive pulmonary disease, or bronchiectasis. NMV-r and MOV were effective in preventing COVID-19-related mortality. The adjusted risk ratio was 0.37 (95% CI: 0.14–1.01, *P* = .05) for NMV-r and 0.42 (95% CI: 0.23–0.77, *P* < .01) for MOV.^[22]^ However, our study specifically focused on only patients with asthma and non-severe COVID-19, with the majority of participants being White. All these findings demonstrated clinical benefits from oral antivirals based on real-world data, reinforcing the use of these antivirals for this particular patient group.

In this study, the effects of NMV-r and MOV were assessed in the subgroup analysis; however, only NMV-r was associated with significant clinical benefit. This result aligns with previous studies suggesting the superiority of NMV-r over MOV in improving clinical outcomes. A systematic review and meta-analysis comparing the efficacy and safety of NMV-r and MOV in treating COVID-19 included 18 studies involving 57,659 patients. The meta-analysis revealed a significant difference between NMV-r and MOV regarding all-cause mortality (odds ratio: 0.54, 95% CI: 0.44–0.67) and all-cause hospitalization (odds ratio: 0.61, 95% CI: 0.54–0.69) rates.^[23]^ Another retrospective study conducted in Hong Kong compared the efficacy of NMV-r and MOV against COVID-19 in unvaccinated adult patients with chronic respiratory diseases. NMV-r was more effective in reducing 90-day mortality with adjusted HR of 0.508 (95% CI: 0.314–0.822, *P* = .006) and shortening the length of stay in the hospital.^[24]^ All these findings indicated that NMV-r should be the first-line drug for this clinical entity, and MOV could be an alternative oral antiviral agent.

According to the guidelines of the Infectious Diseases Society of America,^[14]^ oral antivirals should be administered to patients at high risk of developing severe COVID-19. Although previous studies have identified asthma as a risk factor for disease progression,^[9–13]^ and these patients should be given oral antivirals,^[14]^ the guideline compliance was poor. In this study, the control group had about 5 times more patients than the study group, indicating that most patients with asthma were not treated with oral antivirals in real-world settings. Various factors could influence whether a patient receives antiviral treatment, including rural versus urban location, cost, education, health literacy, patient choice, drug availability, and physician preference. However, the TriNetX database does not provide information on these specific factors, limiting our ability to determine the exact reasons why patients in the control group did not receive oral antivirals. Therefore, more effort is needed to survey the causes of this treatment gap and further enhance compliance with anti-COVID-19 treatment guidelines.

This study has some strengths, including its large sample size and the use of PSM to control for confounding variables. Additionally, we applied an exclusion criterion to exclude patients admitted within 5 days of the index event to ensure that the patients were diagnosed with non-severe COVID-19 initially. We also excluded individuals diagnosed with chronic obstructive pulmonary disease to ensure that only patients with pure asthma were analyzed.

However, this study has some limitations. First, its retrospective nature, based on electronic medical records data, presents certain limitations, such as the potential for entry errors and data gaps. In addition, information about the Charlson Comorbidity Index and detail socioeconomic status were not available. Second, the severity of COVID-19 was not available from the TriNetX network; to focus on non-severe cases, we applied specific exclusion criteria: we removed COVID-19 patients who required hospitalization within 5 days of diagnosis or who died on the day of diagnosis. This approach helps ensure our study population primarily consists of non-severe COVID-19 cases. Third, we could not identify asymptomatic patients with COVID‐19 who did not undergo testing. Fourth, although PSM has been used to balance patients with and without oral antivirals in many respects, some residual confounding factors may still exist. Fifth, although the severity of asthma was determined based on ICD‐10‐CM codes, this method may not be entirely accurate, making it difficult to confidently define asthma severity or control. In this study, we conducted additional sensitivity test on a subgroup of patients requiring triple therapy (ICS/LABA/LAMA), showing the consistent findings. Lastly, the non-significant risk reduction observed in the subgroup of patients with 2 vaccinations or severe asthma may be due to the small number of patients in these subgroups. Further large-scale studies are warranted.

In conclusion, this large retrospective cohort study demonstrated that oral antivirals reduce the risk of adverse outcomes in patients with asthma and non-severe COVID-19 comorbidities. Consistent risk reduction was also observed in subgroups including both sexes, different ages, those with hypertension, diabetes mellitus, dyslipidemia and those who had received less than 1 dose or more than 3 doses of vaccine. Regarding the antiviral agents, NMV-r was associated with notable clinical benefits, whereas MOV was not. This study underscores the importance of oral antivirals, particularly for NMV-r, in managing COVID-19 in patients with asthma and calls for improved compliance with antiviral treatment guidelines to protect this vulnerable population better.

## Acknowledgments

We express our gratitude to Bin Zhang for his consultation on statistical analysis.

## Author contributions

**Conceptualization:** Shao-Chi Fang, Chien-Ming Chao, Chih-Cheng Lai.

**Data curation:** Shao-Chi Fang, Ya-Wen Tsai.

**Formal analysis:** Shao-Chi Fang, Ya-Wen Tsai.

**Investigation:** Shao-Chi Fang, Ya-Wen Tsai, Chien-Ming Chao, Chih-Cheng Lai.

**Methodology:** Shao-Chi Fang.

**Supervision:** Chien-Ming Chao.

**Writing – original draft:** Shao-Chi Fang, Chien-Ming Chao.

**Writing – review & editing:** Chien-Ming Chao, Chih-Cheng Lai.
